# Cytochrome P450 26A1 modulates natural killer cells in mouse early pregnancy

**DOI:** 10.1111/jcmm.13013

**Published:** 2016-11-17

**Authors:** Chao‐Yang Meng, Zhong‐Yin Li, Wen‐Ning Fang, Zhi‐Hui Song, Dan‐Dan Yang, Dan‐Dan Li, Ying Yang, Jing‐Pian Peng

**Affiliations:** ^1^ State Key Laboratory of Stem Cell and Reproductive BiologyInstitute of ZoologyChinese Academy of SciencesBeijingChina; ^2^ University of Chinese Academy of SciencesBeijingChina

**Keywords:** implantation, CYP26A1, natural killer cells, chemokines

## Abstract

Cytochrome P450 26A1 (CYP26A1) has a spatiotemporal expression pattern in the uterus, with a significant increase in mRNA and protein levels during peri‐implantation. Inhibiting the function or expression of CYP26A1 can cause pregnancy failure, suggesting an important regulatory role of CYP26A1 in the maintenance of pregnancy. However, little is known about the exact mechanism involved. In this study, using a pCR3.1‐cyp26a1 plasmid immunization mouse model and a Cyp26a1‐MO (*Cyp26a1*‐specific antisense oligos) knockdown mouse model, we report that the number of *Dolichos biflorus* agglutinin (DBA) lectin‐positive uterine natural killer (uNK) cells was reduced in pCR3.1‐cyp26a1 plasmid immunized and Cyp26a1‐MO‐treated mice. In contrast, the percentage of CD3^−^ CD49b^+^ NK cells in the uteri from the treatment group was significantly higher than that of the control group in both models. Similarly, significantly up‐regulated expression of CD49b (a pan‐NK cell marker), interferon gamma, CCL2, CCR2 (CCL2 receptor) and CCL3 were detected in the uteri of pCR3.1‐cyp26a1‐ and Cyp26a1‐MO‐treated mice. Transcriptome analysis suggested that CYP26A1 might regulate NK cells through chemokines. In conclusion, the present data suggest that silencing CYP26A1 expression/function can decrease the number of uNK cells and significantly increase the percentage of CD3^−^ CD49b^+^ NK cells in the uteri of pregnant mice. These findings provide a new line of evidence correlating the deleterious effects of blocking CYP26A1 in pregnancy with the aberrant regulation of NK cells in the uterus.

## Introduction

Cytochrome P450 26A1 (CYP26A1), an enzyme that metabolizes retinoic acid (RA), is a member of the cytochrome P450 superfamily 1 and is spatiotemporally expressed in the mouse and rat uterine luminal epithelium and glandular epithelium during the peri‐implantation period 2, 3, 4. The human and mouse CYP26A1 proteins exhibit a high degree of amino acid identity (89%) 5. The CYP26A1 mRNA level in the endometrial tissues of premenopausal women was approximately 20‐fold higher in the secretory phase than in the proliferative phase. In addition, the CYP26A1 mRNA level in premenopausal endometria was more than 10‐fold higher than that in postmenopausal endometriosis 6. However, in women with moderate or severe endometriosis, CYP26A1 was significantly down‐regulated in both the early secretory and midsecretory endometrium relative to controls 7, suggesting that CYP26A1 has important functions in both uterine physiology and pathology. The number of implantation sites was markedly reduced by the intrauterine injection of Cyp26a1‐MO (*Cyp26a1*‐specific antisense oligos) or an anti‐CYP26A1 antibody into pregnant mice 3. This finding strongly suggests that uterine CYP26A1 has a critical function in the process of blastocyst implantation.

Uterine natural killer (uNK) cells (the most abundant leucocytes in the early decidua) are transiently present in the uteri of many species and can facilitate endometrial remodelling, angiogenesis and placental formation during pregnancy 8, 9, 10. In the uteri of pregnant mice, uNK cells are present in two specific microdomains, the decidua basalis and the mesometrial lymphoid aggregate of pregnancy (also termed metrial gland) 10. *Dolichos biflorus* agglutinin (DBA) lectin, which has high selectivity for glycoconjugates containing *N*‐acetylgalactosamine in the terminal position, is used as a unique uNK cell marker in mice uteri 11. However, increasing evidence suggests that natural killer (NK) cells play a critical role in foetal resorption, as the *in vivo* depletion of NK cells by anti‐asialoGM1 antibody can reduce abortion rates 12. CD49b (DX5, α_2_ integrin chain), which is expressed by mature NK cells, is widely used as a pan‐NK cell marker in mice 13. CD3^−^ CD49b^+^ NK cells exhibit strong cytotoxicity that can induce pregnancy failure 10, 14. Thus, uNK cells and CD3^−^ CD49b^+^ NK cells have different effects on the process of pregnancy.

CYP26A1 has a critical function in peri‐implantation. However, the exact mechanism by which CYP26A1 affects blastocyst implantation is unclear. At first, we speculated that CYP26A1 exerted its effect *via* degrading RA. Previous experimental results showed that CYP26A1‐regulated Th17 cells were dependent on *all‐trans‐*RA (at‐RA) signalling, which was delivered through RARα during the mouse peri‐implantation period 1. Regrettably, our recent data on the administration RA receptor inhibitors, exogenous RA or RA synthase inhibitors suggested that at‐RA did not affect blastocyst implantation. Consequently, CYP26A1‐regulated Th17 cells, which are dependent on the RARα pathway, played a limited role at the implantation sites. CYP26A1 may be involved in other regulatory pathways in the uterus.

In this study, we found that inhibition of CYP26A1 could significantly modulate the uNK cells and CD3^−^ CD49b^+^ NK cells in the uterus. To our knowledge, this is a novel regulatory pathway and the first evidence demonstrating that CYP26A1, a metabolic enzyme, influences immune tolerance at the maternal‐foetal interface *via* regulating NK cells.

## Materials and methods

### Mice

Eight‐to‐ten‐week‐old healthy female and male BALB/c mice were purchased from SPF (Beijing) Laboratory Animal Technology Co., Ltd. (Beijing, China). The mice were housed in a temperature‐ and humidity‐controlled room with a 12‐hr light/dark cycle and fed standard mouse chow and water. All animal manipulation procedures were approved by the Institutional Animal Care and Use Committee of the Institute of Zoology, Chinese Academy of Sciences (Beijing, China). Female mice were caged overnight with male mice of the same strain at a 2:1 ratio, and the presence of a vaginal plug on the next morning was considered gestational day 1 (GD1).

### Construction of recombinant plasmid and mouse immunization

The plasmid was constructed, and the mice were immunized as previously described with minor modifications 1, 3. Full‐length rat *Cyp26a1* cDNA (94% homology with the mouse *Cyp26a1* cDNA sequence 3) was cloned from the uteri of pregnant rats, and specific primers with *Hindlll/Xhol* restriction sites (forward primer: 5′‐CGAAGCTT (*Hindlll*) ATGGGGCTCCCGGCGCTGCT‐3′; reverse primer: 5′‐CGCTCGAG (*Xhol*) TCAGATATCTCCCTGGAAGTGG‐3′) were utilized. The PCR products were purified and cloned into the pGEM‐T vector (Promega, Madison, WI, USA). Both pGEM‐T‐cyp26a1 and the pCR3.1 vector (Invitrogen, Eugene, OR, USA) were incubated with *Hindlll/Xhol* (Promega) at 37°C for 2 hrs, and then the fragment was ligated into pCR3.1 using T4 ligase (Promega) at 16°C overnight to construct pCR3.1‐cyp26a1. The pCR3.1‐cyp26a1 recombinant plasmid was incubated with *Hindlll/Xhol* at 37°C for 2 hrs, and the inserted fragment was sequenced to determine the accuracy of the sequence. The expression of the recombinant plasmid was detected as previously described with some modifications 15.

The female mice were divided into two groups. One group was immunized with 100 μl saline containing 50 μg pCR3.1‐cyp26a1 per mouse as the treatment group, and the other group was immunized with 100 μl saline containing 50 μg pCR3.1 per mouse as the control group. All the mice were immunized by injecting the plasmid into the thigh muscle. Twenty‐four hours before immunization, each mouse was injected with 100 μl of 0.25% bupivacaine as an adjuvant in the same way. Immunization was performed every 7 days for a total of three times. On the fourth day after the last immunization, the female mice were coupled with male mice at a ratio of 2:1. All the female mice were completely coupled within 3 weeks. All the pregnant mice were killed on GD6 or GD7. Peripheral blood was collected for further analysis. The uteri were excised and divided into fragments. One section was taken for flow cytometry analysis, and the other section was frozen in liquid nitrogen for further analysis.

### Treatment of early pregnant mice with MOs

Morpholino antisense oligonucleotides (MO) were administered by intrauterine injection as previously described with some modifications 3, 16. The following MOs were used (synthesized by Gene Tools, Philomath, OR, USA): Cyp26a1‐MO (MO), 5′‐CATGGCACGCTTCAGCCTCCCGCGC‐3′; standard control MO (Std‐MO), 5′‐CCTCTTACCTCAGTTACAATTTATA‐3′. The MOs were prepared at a stock concentration of 4 mM. The surgery was performed at 8:30 a.m. on GD4. Seven and one‐half μL of a solution containing 30 nmol of Cyp26a1‐MO was injected into the uterine horn of each mouse as the MO treatment group. The same volume of standard control MO was injected into the uterine horn of each mouse as the Std‐MO control group. All the pregnant mice were killed on GD6 or GD7. The injected uteri were excised and divided into fragments. One section was taken for flow cytometry analysis, and the other section was frozen in liquid nitrogen for further analysis.

### RNA isolation, library construction and RNA sequencing

Uteri from the Cyp26a1‐MO knockdown mouse model were used as the material for RNA isolation, library construction and RNA sequencing (RNA‐Seq). Total RNA was prepared with Trizol reagent (Invitrogen) according to the manufacturer's protocol. The total RNA concentration and purity of each sample were measured using a NanoDrop 2000c UV‐Vis Spectrophotometer (Thermo Fisher Scientific Inc., Waltham, Massachusetts, USA), and the quality of the RNA samples was assessed using agarose gel electrophoresis and an Agilent 2100 Bioanalyzer (Agilent Technologies, California, USA). Then, cDNA libraries were constructed and sequenced by Shanghai Majorbio Bio‐Pharm Technology Co., Ltd. (Shanghai, China).

### Total RNA isolation and quantitative PCR

Total RNA was extracted from the uteri using a kit (BioTeke, Beijing, China) and then was reverse transcribed into cDNA using M‐MLV reverse transcriptase (Promega). cDNA was amplified using SYBR Green Master Mix (ComWin Biotech Co. Ltd, Beijing, China) according to the manufacturer's instructions. Quantitative PCR was performed with a LightCycler 480 (Roche, Indianapolis, IN, USA). The target gene mRNA expression was normalized to glyceraldehyde‐3‐phosphate dehydrogenase (GAPDH) expression. The fold change was calculated as 2^−ΔΔCt^ (cycle threshold). The primers used for quantitative PCR are summarized in Table S1.

### Western blotting

Mice uterine proteins were extracted with non‐denaturing lysis buffer (Applygen, Beijing, China), and the protein concentration was examined using a Bicinchoninic Acid Protein Assay Kit (Pierce, Rockford, IL, USA). Uterine proteins were separated by 10% SDS‐PAGE and transferred onto a nitrocellulose membrane (Pall, New York, NY, USA). The membranes were blocked with 10% bovine serum albumin (BSA) in TBST at 37°C for 1 hr and then incubated with the primary antibodies at 4°C overnight. The following primary antibodies were used: rabbit anti‐CD49b (ab133557; Abcam, Cambridge, UK) and rabbit anti‐GAPDH (Boster, Wuhan, China). The membranes were washed and incubated with goat anti‐rabbit secondary antibody conjugated with horseradish peroxidase (HRP) (KPL, Gaithersburg, MD, USA) at 37°C for 1 hr. Then, the membranes were washed. Chemiluminescence reactions were performed with an ECL Detection Kit (Pierce), and images were acquired using Kodak X‐Omat film (Carestream, Xiamen, China). Relative protein levels were analysed using Bio‐Rad Quantity One software (Bio‐Rad, Hercules, CA, USA) and were normalized to GAPDH.

### Immunohistochemistry

The frozen uterine sections (10 μm) were mounted on 3‐aminopropyltriethoxysilane‐coated slides and fixed in 4% paraformaldehyde (PFA) for 15 min. After washing the sections, the sections were blocked with 3% hydrogen peroxide for 10 min. and then 10% normal horse serum (ZSGB‐BIO, Beijing, China) at 37°C for 1 hr. Subsequently, the sections were incubated with rabbit anti‐CD49b (ab133557; Abcam) or rabbit anti‐CCL2 (ab7202; Abcam) primary antibody at 4°C overnight. Then, the sections were incubated with a goat anti‐rabbit secondary antibody conjugated to HRP at 37°C for 1 hr. Direct immunohistochemistry, which involved direct incubation with an antimouse secondary antibody conjugated to HRP, was performed as previously described 15. The colour was developed using a diaminobenzidine tetrahydrochloride (DAB) detection kit (ZSGB‐BIO). Then, sections were counterstained with haematoxylin (Sigma‐Aldrich, St. Louis, MO, USA). The sections were washed with deionized water and dehydrated in graded ethanol solutions followed by xylene. Finally, the sections were mounted with Permount^™^ Mounting Medium. Images were acquired using a Nikon ECLIPSE Ni‐U microscope and NIS software (Nikon, Tokyo, Japan).

### Immunofluorescence

Human breast cancer cell line (MCF‐7) cells, which express CYP26A1 protein, were cytospun onto slides. Then, the slides and sections (10 μm) of pregnant uteri were fixed in 4% PFA for 15 min. The slides were permeabilized in 0.3% Triton X‐100 (Sigma‐Aldrich) for 15 min. After being washed in PBS, the sections and the slides were blocked with 10% normal horse serum at 37°C for 1 hr. Then, the sections were incubated with biotinylated‐DBA lectin (Sigma‐Aldrich), and the slides were incubated with immunized mouse serum at 4°C overnight. Then, the sections were incubated with streptavidin‐FITC, and the slides were incubated with antimouse secondary antibody conjugated to FITC at 37°C for 1 hr. After staining, the sections and slides were washed in PBS and then mounted with antifade mounting media containing propidium iodide (Sigma‐Aldrich). Images were acquired using a confocal microscope (Zeiss LSM 780, Oberkhorn, Germany) or a Nikon ECLIPSE Ni‐U microscope (Nikon).

### Cell suspension preparation and flow cytometry analysis

Uteri were dissected and minced into small fragments. Then, the minced uteri were placed in HBSS containing 200 U/ml hyaluronidase (Sigma‐Aldrich), 1 mg/ml collagenase type IV (Sigma‐Aldrich) and 0.2 mg/ml DNase (Sigma‐Aldrich) and incubated at 37°C for 30 min. as previously described with minor modifications 10. After digestion, the cells were centrifuged and washed with PBS containing 0.2% BSA and incubated in the same buffer for 15 min. at 37°C before filtration through 37 μm nylon mesh. After centrifugation, the cells were resuspended in PBS containing 0.2% BSA for further staining. The cell suspensions were blocked with antimouse CD16/CD32 and then incubated with fluorescently labelled antibody at 4°C for 30 min. The following antibodies were used for flow cytometry analysis: anti‐CD45 PerCP‐Cyanine 5.5 (45‐0451; eBioscience, San Diego, CA, USA), anti‐CD3 PE (12‐0031; eBioscience), anti‐CD49b FITC (11‐0491; eBioscience). After staining, the cells were washed and suspended in PBS containing 0.2% BSA for analysis on a FACScalibur (BD Biosciences, Franklin Lakes, NJ, USA) instrument. The data were analysed using FCS Express V3 software (De Novo Software, Glendale, CA, USA).

### Statistical analysis

Data are presented as the mean ± S.E.M. All the results were analysed using a paired *t*‐test to assess the significance of the differences. A value of *P* < 0.05 was considered statistically significant, and a value of *P* < 0.01 was considered sufficient statistical significance. Statistical analysis was performed with SPSS version 16.0 software (SPSS Software, Chicago, IL, USA).

## Results

### The number of implantation sites is reduced in pCR3.1‐cyp26a1‐ and Cyp26a1‐MO‐treated mice

**Figure 1:**
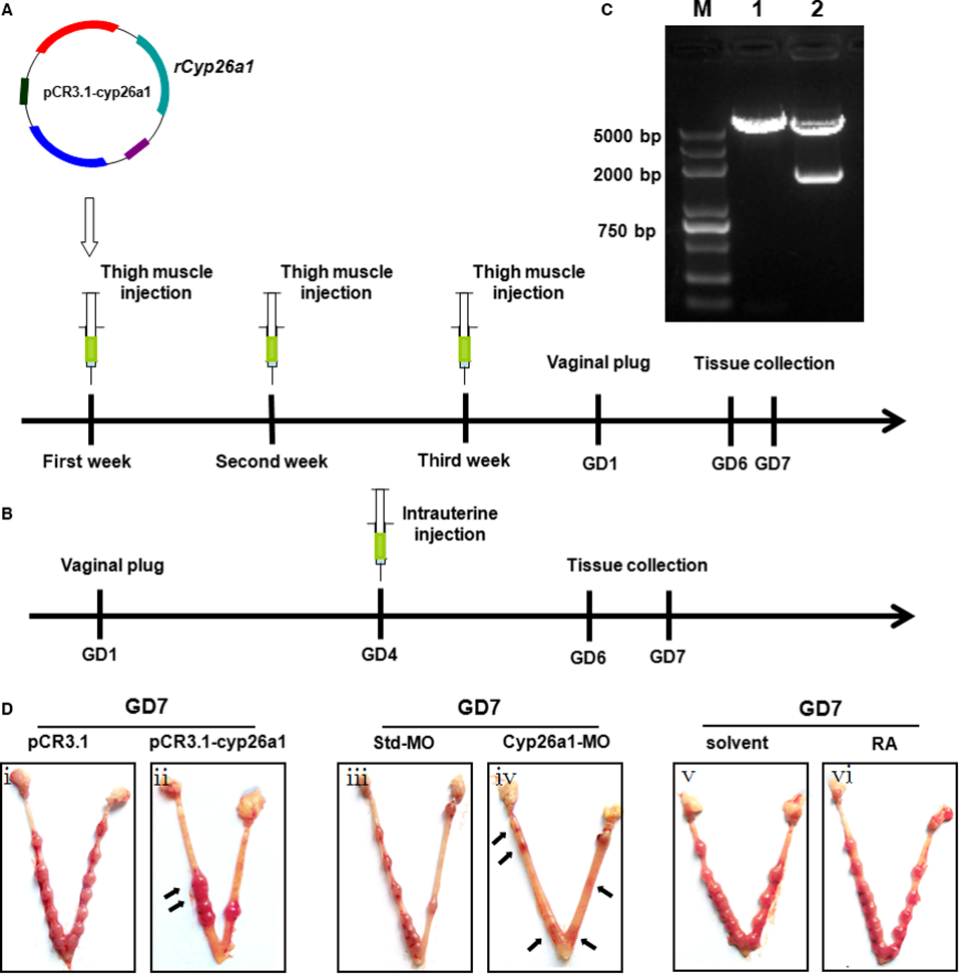
Schematic illustration of the experimental models. (**A**) Schematic illustration of the pCR3.1‐cyp26a1 plasmid immunization mouse model. pCR3.1‐cyp26a1 or pCR3.1 was injected into the thigh muscle of female mice every 7 days for a total of three times. Mouse uteri were collected on GD6 or GD7. (**B**) Schematic illustration of the Cyp26a1‐MO knockdown mouse model. Cyp26a1‐MO or Std‐MO was injected into the uterine lumen on GD4. Mouse uteri were collected on GD6 or GD7. (**C**) Identification of recombinant plasmid pCR3.1‐cyp26a1 by restriction digestion. M: DNA marker; 1: pCR3.1 without rat *Cyp26a1* insertion was digested by *Hindlll/Xhol*; 2: pCR3.1‐cyp26a1 was digested by *Hindlll/Xhol*. Rat *Cyp26a1* cDNA had a length of approximately 1500 bp. (**D**) Representative macroscopic views of the uteri of control mice after pCR3.1, Std‐MO or solvent injection (**i**,** iii** and **v**) and the uteri of treated mice after pCR3.1‐cyp26a1, Cyp26a1‐MO or RA injection (**ii**,** iv** and **vi**) are shown. Arrows indicate the abnormal embryos.

Schematic illustrations of the pCR3.1‐cyp26a1 plasmid immunization mouse model and the Cyp26a1‐MO knockdown mouse model are shown in Figure 1A and B respectively. The pCR3.1‐cyp26a1 recombinant plasmid was evaluated with restriction digestion. Rat *Cyp26a1* cDNA had a length of approximately 1500 bp (Fig. 1C). The uteri of control mice (pCR3.1, Std‐MO) exhibited grossly morphologically normal implantation sites (Fig. 1Di and iii). In contrast, the number of implantation sites was reduced in the uteri of treated mice (pCR3.1‐cyp26a1, Cyp26a1‐MO) (Fig. 1Dii and iv). Furthermore, in the pCR3.1‐cyp26a‐treated mice, some embryos were abnormal (Fig. 1Dii). In the Cyp26a1‐MO‐treated mice, we found that the resorption of the uterine contents was characterized by the degeneration of the decidual tissue accompanied by thrombosis and haemorrhage. The remnants of the decidua had already passed into the uterine lumen with the embryos (Fig. 1Div). The number of implantation sites was not statistically different in the mice administered exogenous RA on GD4 and the control mice (Fig. 1Dv and vi).

### Mice immunized with the pCR3.1‐cyp26a1 plasmid produce anti‐CYP26A1 antibodies

**Figure 2:**
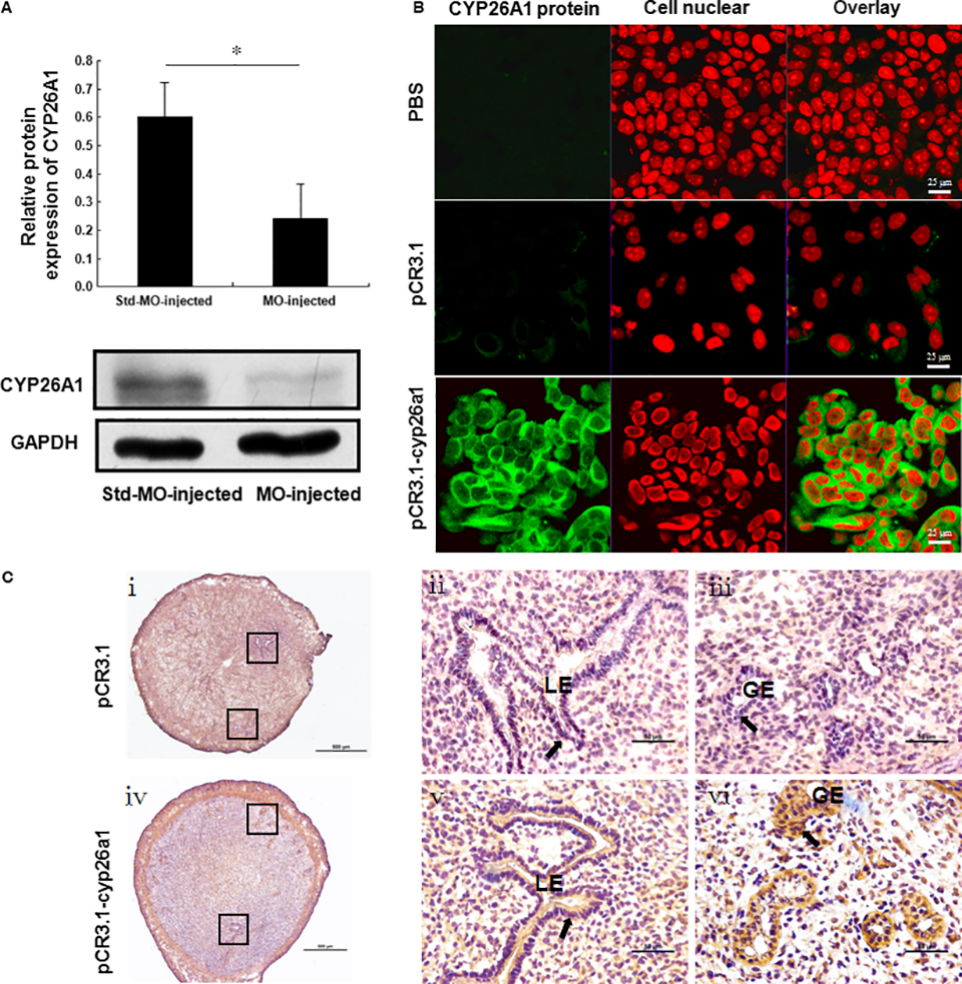
Validation of the pCR3.1‐cyp26a1 plasmid immunization mouse model and the Cyp26a1‐MO knockdown mouse model. Mated BALB/c female mice from the two models were sacrificed on GD7. (**A**) CYP26A1 expression in the uteri of the Cyp26a1‐MO knockdown mouse model was analysed with western blotting. The data are presented as the mean ± S.E.M. of four independent experiments and were obtained from four mice in each group. *P < 0.05 by independent samples *t*‐test. (**B**) The expression of anti‐CYP26A1 antibodies in the pCR3.1‐cyp26a1 plasmid immunization mouse model was analysed with immunofluorescence. MCF‐7 cells were reacted with the sera of mice immunized with PBS, pCR3.1 and pCR3.1‐cyp26a1. The green signals represent the CYP26A1 protein in MCF‐7 cells binding with the anti‐CYP26A1 antibody in the serum, and the red signals represent the nuclei of the cells. The photomicrographs are representative of three mice in each group, scale bar: 25 μm. (**C**) The expression of anti‐CYP26A1 antibodies by the pCR3.1‐cyp26a1 plasmid immunization mouse model was analysed by direct immunohistochemistry. Arrows show that positive signals is observed in the LE and GE of the uteri from pCR3.1‐cyp26a1 immunized mice. Panels **ii**,** iii** and **v**,** vi** are higher magnifications of the different areas marked with the black rectangles in panels **i** and **iv** respectively. Photomicrographs are representative of at least three mice in each group, scale bar: 500 μm (**i** and **iv**) and 50 μm (**ii**,** iii**,** v** and **vi**). GE: glandular epithelium; LE: luminal epithelium.

To examine the production of anti‐CYP26A1 antibodies by the mice immunized with the pCR3.1‐cyp26a1 plasmid, we performed immunofluorescence and direct immunohistochemistry. The serum samples from the mice immunized with pCR3.1 or pCR3.1‐cyp26a1 on GD7 were analysed with immunofluorescence. As show in Figure 2B, the green fluorescence of the serum from the mice immunized with pCR3.1‐cyp26a1 was higher than that from the control mice immunized with pCR3.1 or PBS. Furthermore, direct immunohistochemistry analysis indicated positive signals in the luminal epithelium and glandular epithelium of the uteri from pCR3.1‐cyp26a1 immunized mice compared with those from the pCR3.1 immunized mice (Fig. 2C). The above results demonstrated that the mice immunized with pCR3.1‐cyp26a1 produced anti‐CYP26A1 antibodies.

### CYP26A1 expression is reduced in the uteri of Cyp26a1‐MO‐treated mice

To analyse the efficacy of Cyp26a1‐MO in inhibiting the production of CYP26A1 protein, western blotting was performed to detect the expression of the CYP26A1 protein in the uteri. The expression of the CYP26A1 protein was significantly decreased in Cyp26a1‐MO‐treated mice on GD7 (Fig. 2A). This result confirmed that the production of CYP26A1 protein was inhibited by the specific MO.

### The number of uNK cells is decreased in pCR3.1‐cyp26a1 and Cyp26a1‐MO‐treated mice

**Figure 3:**
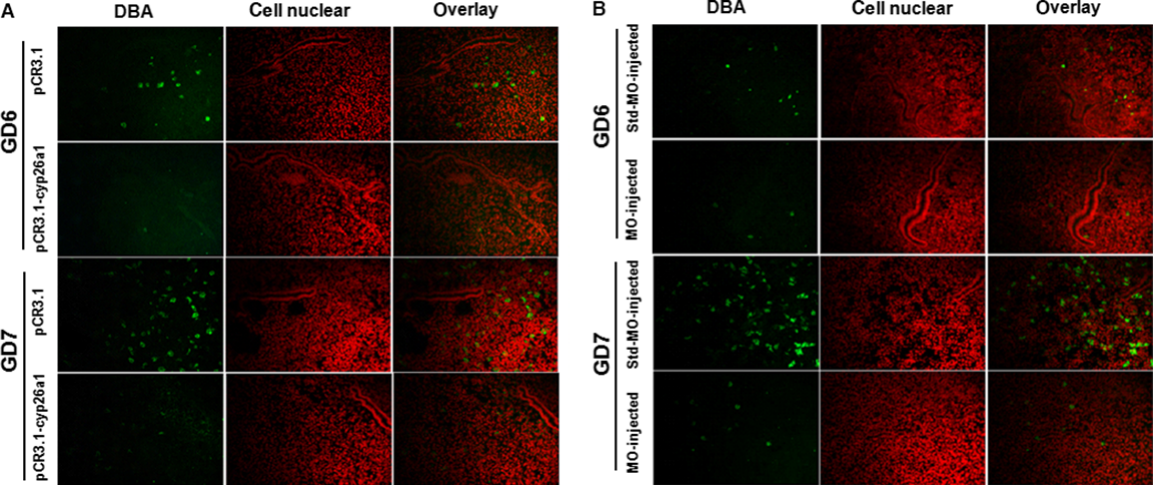
pCR3.1‐cyp26a1 or Cyp26a1‐MO administration decreases the number of uNK cells. Mated BALB/c female mice from the two models were killed on GD6 or GD7. (**A**) Immunofluorescence analysis of DBA lectin‐stained uNK cells in the uteri from the pCR3.1‐cyp26a1 plasmid immunization mouse model. The green signals represent DBA lectin‐positive cell staining, and the red signals indicate nuclear staining. Stronger positive staining is observed in the DB of the uteri from pCR3.1 immunized mice (the control group) on GD6 and GD7. The photomicrographs show 200× original magnification and are representative of three mice in each group. (**B**) Immunofluorescence analysis of DBA lectin‐stained uNK cells in the uteri of the Cyp26a1‐MO knockdown mouse model. The green signals represent DBA lectin‐positive cell staining, and the red signals indicate nuclear staining. Stronger positive staining is observed in the DB of the uteri from Std‐MO‐treated mice (the control group) on GD6 and GD7. The photomicrographs show 200× original magnification and are representative of three mice in each group. DB: decidua basalis.

Uterine natural killer cells have important functions that are necessary for the progression of pregnancy 17. We analysed whether pCR3.1‐cyp26a1 and Cyp26a1‐MO treatment would alter the uNK cells. By performing immunofluorescence analysis, we discovered that the DBA lectin‐positive uNK cells were restricted to the decidua basalis in mouse uteri (Fig. 3A and B) and that there were fewer DBA lectin‐positive uNK cells on GD6 and GD7 in the treated mice than in the control mice in both models (Fig. 3A and B). This result strongly indicated that pCR3.1‐cyp26a1 and Cyp26a1‐MO treatment could reduce the number of uNK cells.

### The percentage of CD3^−^ CD49b^+^ NK cells is increased in the uteri of pCR3.1‐cyp26a1‐ and Cyp26a1‐MO‐treated mice

**Figure 4:**
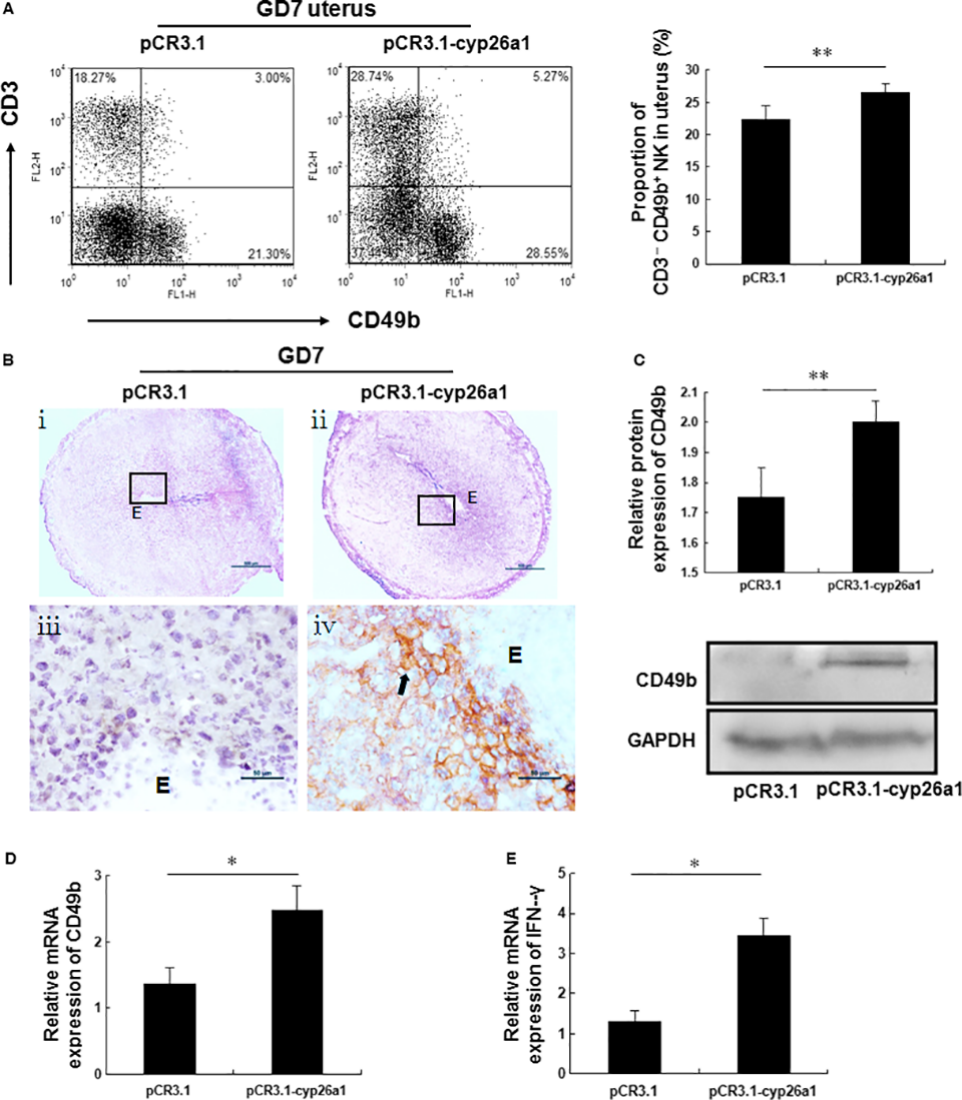
pCR3.1‐cyp26a1 treatment significantly increased the percentage of CD3^−^ CD49b^+^ NK cells in the uteri. Mated BALB/c female mice from the pCR3.1‐cyp26a1 plasmid immunization mouse model were killed on GD7. (**A**) The percentage of CD3^−^ CD49b^+^ NK cells (lower right quadrant) among the CD45^+^ leucocytes in the uteri was analysed with flow cytometry analysis. One representative experiment is shown. See Figure S1 for the gating strategy for CD3^−^ CD49b^+^ NK cells. The right panel shows the statistics for the percentages of CD3^−^ CD49b^+^ NK cells. The data are presented as the mean ± S.E.M. of five independent experiments and were obtained from five mice in each group. **P < 0.01 by independent samples *t*‐test. (**B**–**D**) CD49b expression in the uteri was analysed by immunohistochemistry, western blotting and quantitative PCR. (**B**) The arrow indicates that positive signals is observed in the uteri from pCR3.1‐cyp26a1 immunized mice. Panels **iii** and **iv** are higher magnifications of the different areas marked with the black rectangles in panels **i** and **ii** respectively. Photomicrographs are representative of three mice in each group, scale bar: 500 μm (**i** and **ii**) and 50 μm (**iii** and **iv**). (**C** and **D**) The data are presented as the mean ± S.E.M. of four (western blotting) or four (quantitative PCR) independent experiments and were obtained from four mice each group. *P < 0.05 and **P < 0.01 by independent samples *t*‐test. (**E**) IFN‐γ expression was analysed in the uteri using quantitative PCR. The data are presented as the mean ± S.E.M. of four independent experiments and were obtained from four mice in each group. *P < 0.05 by independent samples *t*‐test. E: embryo.

**Figure 5:**
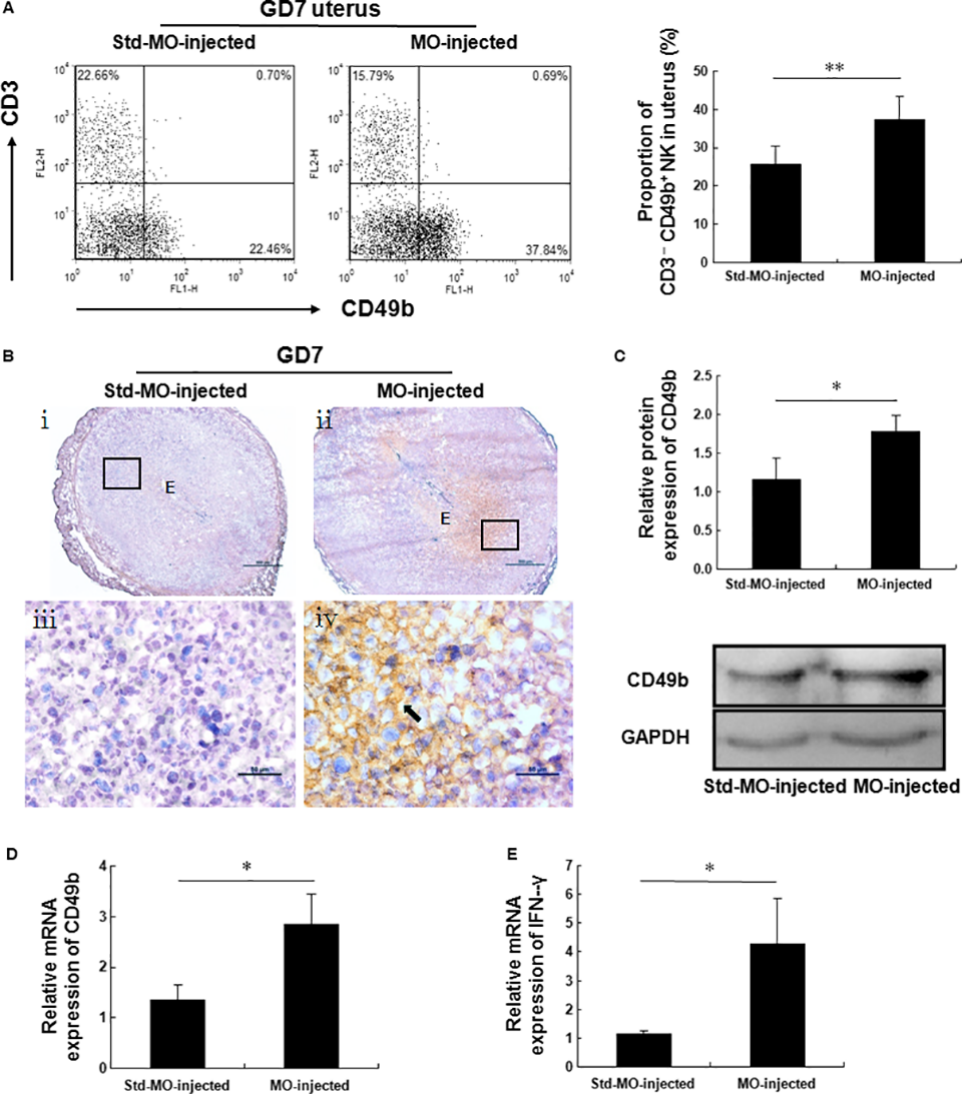
Cyp26a1‐MO treatment significantly increases the percentage of CD3^−^ CD49b^+^ NK cells in the uterus. Mated BALB/c female mice from the Cyp26a1‐MO knockdown mouse model were killed on GD7. (**A**) The percentage of CD3^−^ CD49b^+^ NK cells (lower right quadrant) among the CD45^+^ leucocytes in the uteri was analysed with flow cytometry analysis. One representative experiment is shown. See Figure S1 for the gating strategy for CD3^−^ CD49b^+^ NK cells. The right panel shows the statistics for the percentages of CD3^−^ CD49b^+^ NK cells. The data are presented as the mean ± S.E.M. of five independent experiments and were obtained from five mice in each group. **P < 0.01 by independent samples *t*‐test. (**B**–**D**) CD49b expression in the uteri was analysed with immunohistochemistry, western blotting and quantitative PCR. (**B**) The arrow indicates the positive signals observed in the uteri from Cyp26a1‐MO‐treated mice. Panels **iii** and **iv** are higher magnifications of the different areas marked with the black rectangles in panels **i** and **ii** respectively. Photomicrographs are representative of three mice in each group, scale bar: 500 μm (**i** and **ii**) and 50 μm (**iii** and **iv**). (**C** and **D**) The data are presented as the mean ± S.E.M. of four (Western blotting) or four (quantitative PCR) independent experiments and were obtained from four mice each group respectively. *P < 0.05 by independent samples *t*‐test. (**E**) IFN‐γ expression in the uteri was analysed with quantitative PCR. The data are presented as the mean ± S.E.M. of four independent experiments and were obtained from four mice in each group. *P < 0.05 by independent samples *t*‐test. E: embryo.

Unlike DBA^+^ uNK cells, CD3^−^ CD49b^+^ NK cells exhibit strong cytotoxicity 13, 14 and are incompatible with successful pregnancy 10. We examined whether pCR3.1‐cyp26a1 and Cyp26a1‐MO treatment could alter the percentage of CD3^−^ CD49b^+^ NK cells in the uterus. Uterine cells were collected to evaluate the percentage of CD3^−^ CD49b^+^ NK cells among the CD45^+^ leucocytes (see Fig. S1 for the gating strategy) using flow cytometry analysis. The percentage of CD3^−^ CD49b^+^ NK cells (lower right quadrant) in the uteri from pCR3.1‐cyp26a1‐treated mice was significantly higher than that from the pCR3.1‐treated mice on GD7 (Fig. 4A). By performing quantitative PCR analysis, we found that the expression of CD49b mRNA was markedly up‐regulated by pCR3.1‐cyp26a1 treatment on GD7 (Fig. 4D). The significant increase in CD49b protein expression was also confirmed by western blotting (Fig. 4C). Furthermore, immunohistochemistry analysis showed positive signals in the uteri from pCR3.1‐cyp26a1‐treated mice compared with those from the control mice (Fig. 4B). Interestingly, interferon gamma (IFN‐γ) expression was significantly up‐regulated in the uteri from pCR3.1‐cyp26a1‐treated mice on GD7 (Fig. 4E). In the Cyp26a1‐MO‐treated mice, flow cytometry analysis also revealed that the percentage of CD3^−^ CD49b^+^ NK cells among the CD45^+^ leucocytes in the uteri significantly increased on GD7 (Fig. 5A). Quantitative PCR, western blotting and immunohistochemistry all showed higher CD49b expression in the uteri of Cyp26a1‐MO‐treated mice on GD7 than in the control mice (Fig. 5D, C and B). Similarly, the expression of IFN‐γ mRNA was markedly up‐regulated in the uteri from Cyp26a1‐MO‐treated mice on GD7 (Fig. 5E). Overall, our results strongly revealed that pCR3.1‐cyp26a1 and Cyp26a1‐MO induced a significant increase in the percentage of CD3^−^ CD49b^+^ NK cells in the uteri.

### Transcriptome analysis suggests that CYP26A1 regulates NK cells through chemokines

**Figure 6:**
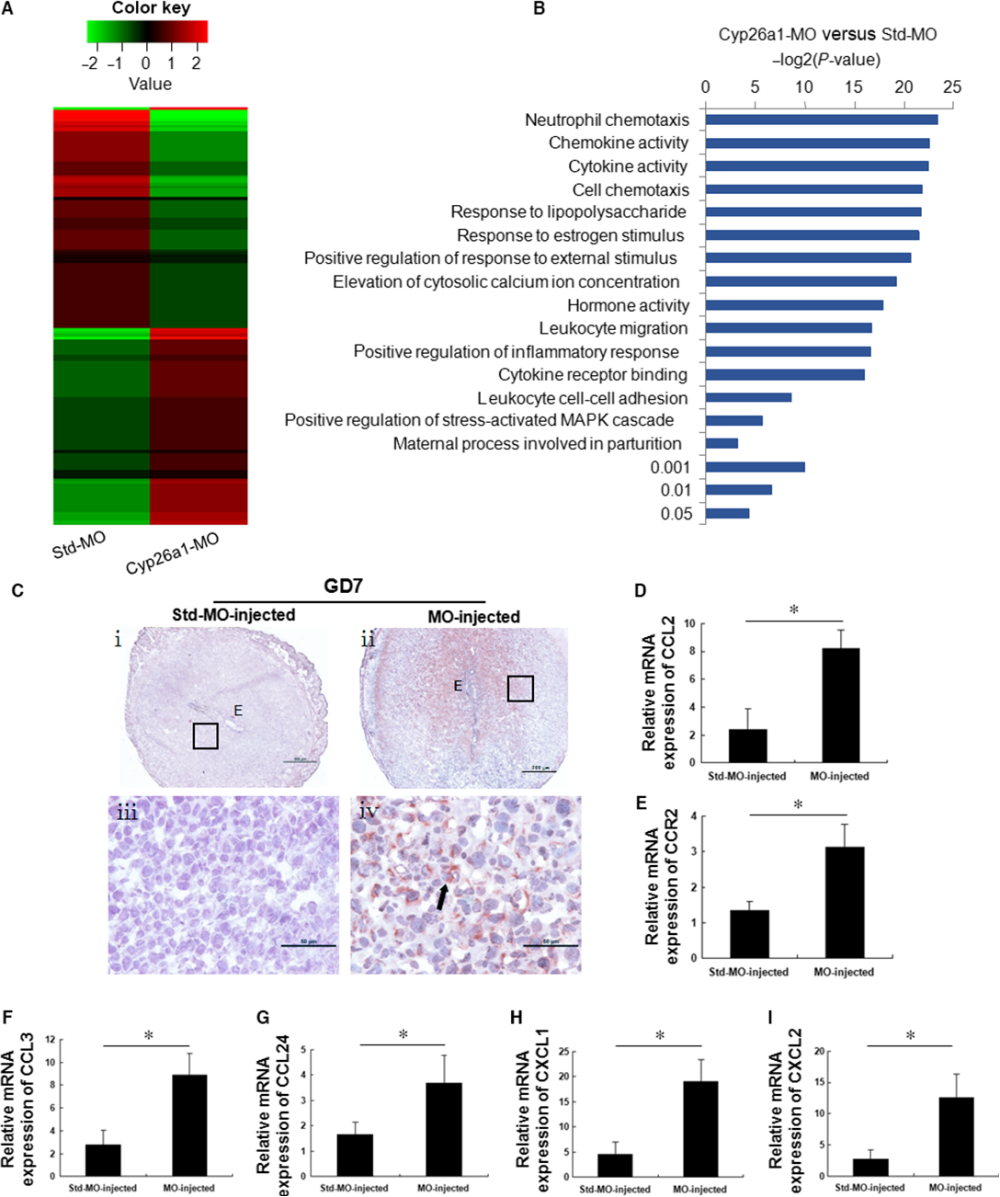
CYP26A1 regulates NK cells through chemokines. Mated BALB/c female mice from the Cyp26a1‐MO knockdown mouse model were killed on GD7. (**A**) The inhibition of CYP26A1 in the uterus leads to distinct transcriptome profiles. The heat maps show the hierarchical clustering of differentially expressed genes (FDR <0.05, |log2FC| >1). (**B**) Bar plot of the GO analysis of the differentially expressed genes in the uteri of Cyp26a1‐MO‐treated mice and control mice. The *y*‐axis shows the GO terms, and the *x*‐axis shows the enrichment significance *P*‐values. (**C**–**I**) CYP26A1 inhibition significantly increases uterine CCL2, CCR2, CCL3, CCL24, CXCL1 and CXCL2 expression. (**C**) CCL2 expression in the uterus was analysed using immunohistochemistry. The arrow indicates the positive signals observed in the Cyp26a1‐MO‐treated mice. Panels **iii** and **iv** are higher magnifications of the different areas marked with the black rectangles in panels **i** and **ii** respectively. The photomicrographs are representative of three mice in each group, Scale bar: 500 μm (**i** and **ii**) and 50 μm (**iii** and **iv**). (**D**–**I**) CCL2, CCR2, CCL3, CCL24, CXCL1 and CXCL2 expression in the uteri was analysed with quantitative PCR. The data are presented as the mean ± S.E.M. of four independent experiments and were obtained from four mice in each group. *P < 0.05 by independent samples *t*‐test. E: embryo.

To further investigate how CYP26A1 regulates NK cells, RNA‐Seq was performed to detect the differentially expressed genes in the uteri of Cyp26a1‐MO‐treated mice and control mice. Transcriptome analysis revealed that 355 unigenes were differentially expressed (FDR <0.05, |log2FC| >1) in the uteri of the Cyp26a1‐MO‐treated mice and the control mice; of these, 177 unigenes were significantly up‐regulated and 178 unigenes were significantly down‐regulated. Heat maps showed the hierarchical clustering of differentially expressed genes (Fig. 6A). The Kyoto Encyclopedia of Genes and Genomes enrichment analysis showed that most enrichment pathways focused on MHC‐induced immune responses in the uteri. Furthermore, Gene Ontology (GO) analysis of the differentially expressed genes in the uteri indicated that chemokine activity, cell chemotaxis and leucocyte migration were significantly enriched (Fig. 6B). In addition, compared with that in the control mice, the RNA‐Seq data showed that nine chemokines were significantly up‐regulated, and two chemokines were significantly down‐regulated in the uteri of Cyp26a1‐MO‐treated mice (Table 1). These results were confirmed with the quantitative PCR, which revealed significantly elevated mRNA levels of CCL2, CCL3, CCL24, CXCL1 and CXCL2 in the uteri of Cyp26a1‐MO‐treated mice relative to control mice (Fig. 6D and F–I). Natural killer cells express chemokine receptors (including CCR2, CCR5, CX3CR1 and CXCR3) that allow them to participate in diverse types of inflammatory reactions 14, 18, 19. It has been reported that CCL3 regulates the trafficking of mouse NK cells 20. In this study, CCL2 (CCR2 ligand), CCL3 (CCR5 ligand) and CCL8 (CCR2 and CCR5 ligand) expression was significantly up‐regulated in the uteri of Cyp26a1‐MO‐treated mice compared with that in the control mice (Fig. 6D, F and Table 1). The significantly enhanced expression of CCL2 was confirmed by quantitative PCR and immunohistochemistry (Fig. 6C and D). Interestingly, the expression of CCR2 (NK cell receptor) mRNA was markedly up‐regulated by Cyp26a1‐MO treatment on GD7 (Fig. 6E). These results suggested that the interaction of CCL2 and CCL8 with CCR2 might stimulate the chemotaxis of CD3^−^ CD49b^+^ NK cells in the uterus.

**Table 1:** Chemokines were significantly changed in uteri from the Cyp26a1‐MO knockdown mouse model

transcript_id	Chemokines	C_FPKM*	T_FPKM*	log2FC (T/C)†	Up–down‐regulation (T/C)
ENSMUST00000075433	CXCL2	0.0527529	2.45867	5.542483895	Up
ENSMUST00000031327	CXCL1	0.238117	7.14426	4.907042057	Up
ENSMUST00000000193	CCL2	2.07477	14.1313	2.767870872	Up
ENSMUST00000004936	CCL24	0.834129	5.24841	2.653538004	Up
ENSMUST00000031318	CXCL5	1.72974	6.70619	1.954938158	Up
ENSMUST00000026911	CCR1	1.33516	3.5824	1.423913794	Up
ENSMUST00000019266	CCL9	3.02597	7.67749	1.343237044	Up
ENSMUST00000001008	CCL3	0.546739	1.36506	1.32004017	Up
ENSMUST00000009329	CCL8	88.6461	188.97	1.092028151	Up
ENSMUST00000039171	CCR3	3.72182	1.40464	−1.405807859	Down
ENSMUST00000099241	CCL28	5.74531	1.71189	−1.746794736	Down

*C_FPKM and T_FPKM (Fragments per kilobase of exon model per million mapped reads) indicate the normalized numbers of reads for each transcript from the control and treatment samples respectively. ^†^Log2FC (T/C) is the log to base 2 of the difference in transcript abundance between the control and treatment samples.

## Discussion

In this study, inhibiting the function or expression of CYP26A1 decreased the number of uNK cells and significantly increased the percentage of CD3^−^CD49b^+^ NK cells in the uteri of pregnant mice. To our knowledge, this is the first study to correlate CYP26A1 with NK cells *in vivo*.

Our experimental results showed that the administration of exogenous RA on GD4 did not cause the number of implantation sites to differ significantly from that in the control mice. This suggested that RA might not affect or only subtly affect blastocyst implantation, which seems to be consistent with a previous report that RA might have no direct effect on regulating the process of blastocyst implantation 21. CYP26A1, a RA‐metabolizing enzyme, may be involved in other regulatory pathways that affect blastocyst implantation in the uterus. Spatial and temporal immunological shifts are necessary for implantation and the progression of pregnancy 22. Thus, we speculated that CYP26A1 might regulate immune cells in mice during the peri‐implantation period. To test this hypothesis, the pCR3.1‐cyp26a1 plasmid immunization mouse model and the Cyp26a1‐MO knockdown mouse model were developed to study whether CYP26A1 correlated with NK cell activity.

This study was based on two distinct methods of silencing of CYP26A1 expression/function. The first one was based on mouse immunization with the pCR3.1‐cyp26a1 plasmid, and the second one was based on the injection of morpholino antisense nucleotides (Cyp26a1‐MO). The applicability of plasmids in studies on reproduction has been demonstrated 1, 3, 15. Plasmid immunization has more advantages than protein immunization and can induce a long‐lasting antibody response 23, 24. Morpholinos are synthetic DNA analogues with highly favourable properties as *in vivo* gene‐targeting tools 16, 25. It has been reported that MOs were used to effectively disrupt protein expression in developmental systems such as zebrafish embryos and mouse oocytes 26, 27 and to study the blastocyst implantation process 3, 16, 28. Morpholinos can penetrate the mouse uterine luminal epithelium and the underlying stromal cells after intrauterine injection 3, 16. Therefore, we performed intrauterine injection to inhibit the production of the CYP26A1 protein using Cyp26a1‐MO, which was directly injected into the uterine lumen.

Female CBA/J mice mated with DBA/2 males had an abnormal resorption rate that was thought to be due to the activation of NK cells and mononuclear cells expressing Mac‐1 (CD11b) and F4/80 12, 29. Furthermore, human recurrent pregnancy loss is associated with NK cells, and the percentage of CD3^−^ CD49b^+^ NK cells in the uteri of the mouse foetus abortion group was significantly increased 10, 30. Our results showed that the percentage of CD3^−^ CD49b^+^ NK cells in the uteri from pCR3.1‐cyp26a1‐ and Cyp26a1‐MO‐treated mice was markedly increased on GD7. These results seemed to be consistent with previous reports showing that the cytotoxicity of CD3^−^ CD49b^+^ NK cells is stronger than that of CD49b^−^ NK cells 13 and that CD3^−^ CD49b^+^ NK cells are incompatible with successful pregnancy 10. Natural killer cells can secrete IFN‐γ, and IFN‐γ has cytotoxicity and is detrimental to pregnancy 10, 14, 31, 32. Our data revealed that IFN‐γ expression was markedly up‐regulated in the uteri from pCR3.1‐cyp26a1‐ and Cyp26a1‐MO‐treated mice. Thus, CD3^−^ CD49b^+^ NK cells exerted adverse effects on pregnancy, possibly by secreting IFN‐γ. In addition, we observed fewer DBA^+^ uNK cells in the uteri of pCR3.1‐cyp26a1‐ and Cyp26a1‐MO‐treated mice than in the uteri of control mice on GD6 and GD7. Increasing evidence supports the idea that uNK cells make major contributions to induce the remodelling of decidual arteries and vascularization 33. Thus, a lower number of DBA^+^ uNK cells might be disadvantageous to maintaining pregnancy. The DBA^+^ uNK cell population has a unique Ly49 receptor repertoire and an unusual NKp46^+^NK1.1^−^ CD49b^−^ NKG2D^+^ phenotype compared with CD3^−^ CD49b^+^ NK cells. CD3^−^ CD49b^+^ NK cells are only a minor subset during normal pregnancy in mice 9, whereas pCR3.1‐cyp26a1 or Cyp26a1‐MO administration significantly increased the percentage of CD3^−^ CD49b^+^ NK cells in the uteri. Therefore, these results showed that the reduced function or expression of CYP26A1 alters the number of DBA^+^ uNK cells and the percentage of CD3^−^ CD49b^+^ NK cells in the uteri. The different effects of CYP26A1 on DBA^+^ uNK cells and CD3^−^ CD49b^+^ NK cells might be because of their distinct response to the blocking of CYP26A1 *in vivo*.

Chemokines are a group of small, structurally related molecules that coordinate the homoeostatic circulation of leucocytes 34 and stimulate efficient chemotactic activity in monocytes, T cells and NK cells 35. Chemokines may play a fundamental role in generating a specialized immune milieu at the maternal–foetal interface by recruiting immune cells 36, 37, 38. Natural killer cells express chemokine receptors (including CCR2, CCR5, CX3CR1 and CXCR3) that allow them to be recruited to tissues after infection or inflammation 14, 18, 19. Chemokines play a major role in this process. Following activation, NK cells can migrate in response to additional CC and CXC chemokines 18. It has been reported that CCL3, CX3CL1, CXCL10 and CXCL12 regulate the trafficking of mouse NK cells 10, 20, 35. In addition, CCL2 and CCL3 can augment the cytolytic activity of NK cells 18. Our data revealed that CCL2, CCL3 and CCL8 expression was significantly up‐regulated in the uteri of Cyp26a1‐MO‐treated mice relative to control mice. Interestingly, the expression of CCR2 (NK cell receptor) mRNA was markedly up‐regulated in Cyp26a1‐MO‐treated mice. Collectively, these results suggest that CYP26A1 might regulate CD3^−^ CD49b^+^ NK cells through chemokines. However, the exact mechanism still needed further experiments to elucidate in the mice.

Human epidermal keratinocytes express only a low basal level of CYP26A1 but do not exhibit CYP26A1 induction by RA 39. CYP26 RNA is induced by RA in the mouse liver, but RA treatment has no effect on the expression level in the brain 40. In addition, our data revealed that uterine CYP26A1 was not induced by the administration of exogenous RA to the mice (data not shown). These results suggest that CYP26A1 may not be induced by RA in the mouse uterus and has tissue specificity. Furthermore, a previous report mentioned that it cannot be concluded that the CYP26 family exclusively metabolizes RA 41. Moreover, the application of the CYP26A1 antagonist R115866 (a potent and selective inhibitor of at‐RA metabolism 42) can lead to a significant increase in the uterine at‐RA concentration and CYP26A1 mRNA expression in the liver, but CYP26A1 mRNA expression during blastocyst implantation is not significantly different in the uteri of R115866‐treated rats relative to control rats 21. These data suggest that uterine CYP26A1 mRNA expression did not change in response to the increase in uterine at‐RA level induced by R115866 administration. However, uterine CYP26A1 expression has been confirmed as mainly regulated by gestagen 21, which can induce CYP26A1 expression in the uterine endometrial epithelial and glandular cells of mice 43, 44. CYP26A1 induction by gestagen is a physiological pathway, and this pathway may be necessary for the receptivity of the maternal endometrium to blastocyst implantation and later decidualization 21, 44. Collectively, these findings indicate that gestagen can regulate CYP26A1. Thus, the silencing of CYP26A1 can significantly increase the proportion of uterine CD3^−^ CD49b^+^ NK cells through chemokines, and CD3^−^ CD49b^+^ NK cells can secrete IFN‐γ, which is a Th1‐type cytokine and is detrimental to pregnancy. In addition, the silencing of CYP26A1 can reduce the number of uNK cells in the uteri of pregnant mice.

In conclusion, our data suggest that exogenous pCR3.1‐cyp26a1 or Cyp26a1‐MO administration can lead to the aberrant modulation of uNK cells and CD3^−^ CD49b^+^ NK cells in the uteri of mice during peri‐implantation. This is a novel mechanism, in which blocking CYP26A1 can cause pregnancy failure through the regulation of uNK cells and CD3^−^ CD49b^+^ NK cells. These new findings improve our knowledge of the mechanism of blocking CYP26A1 and have potential clinical implications.

## Conflict of interest

The authors confirm that there are no conflicts of interest.

## Author contribution

J‐PP designed the experiments, provided critical reagents and experimental expertise and supervised the study; C‐YM designed the experiments, performed the experiments, analysed the data and wrote the manuscript; Z‐YL, W‐NF, Z‐HS, D‐DY, D‐DL and YY performed some of the experiments; DP analysed some of the data.

## Supporting information


**Figure S1** The flow cytometric gating strategy of CD3^−^ CD49b^+^ NK cells (lower right quadrant) is shown.
**Table S1** Quantitative PCR primers for the detection of mRNA expression.
