# Supplementary material for: Cytochrome P450 26A1 modulates natural killer cells in mouse early pregnancy
Source: J Cell Mol Med. 2016 Nov 17;21(4):697–710. doi: 10.1111/jcmm.13013 (PMC5345621; doi:10.1111/jcmm.13013)
Supplement: Supplementary file 1 — Figure S1 The flow cytometric gating strategy of CD3− CD49b+ NK cells (lower right quadrant) is shown. Table S1 Quantitative PCR primers for the detection of mRNA expression. [file JCMM-21-697-s001.docx]

**Cytochrome P450 26A1 modulates natural killer cells in mouse early pregnancy**

**Chao-Yang Meng ^a, b^, Zhong-Yin Li ^a^, Wen-Ning Fang ^a, b^, Zhi-Hui Song ^a, b^, Dan-Dan Yang ^a, b^, Dan-Dan Li ^a, b^, Ying Yang ^a^, Jing-Pian Peng ^a, *^**

^a^ State Key Laboratory of Stem Cell and Reproductive Biology, Institute of Zoology, Chinese Academy of Sciences, Beijing, China

^b^ University of Chinese Academy of Sciences, Beijing, China

^*^ Correspondence to: Jing-Pian Peng; 1 Beichen West Road, Chaoyang District, Beijing 100101, China; Tel: +86-10-64807183; Fax: +86-10-64807099; E-mail: pengjp@ioz.ac.cn

**Supplementary data**

**
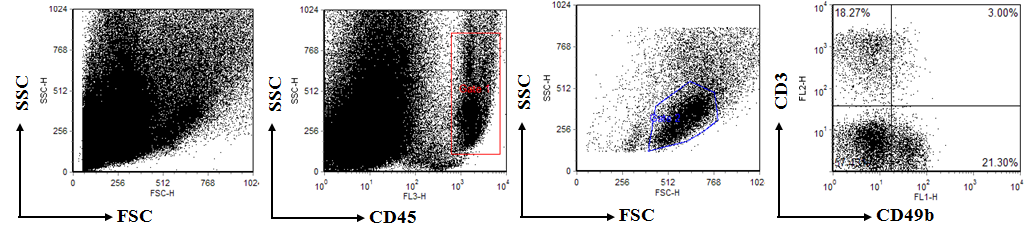
**

**Supplementary Fig. S1** The flow cytometric gating strategy of CD3^−^CD49b^+^ NK cells (lower right quadrant) is shown. Dot plots shown are gating strategy to analyze CD3^−^CD49b^+^ NK cells in the uterus. Pan leucocytes are gated by using anti-CD45 antibody versus SSC and then back gate analysis of CD3^−^CD49b^+^ NK cells is shown through FSC and SSC. CD3^−^CD49b^+^ NK cells are gated by using anti-CD3 and anti-CD49b antibody.

**Supplementary Table S1** Quantitative PCR primers for the detection of mRNA expression


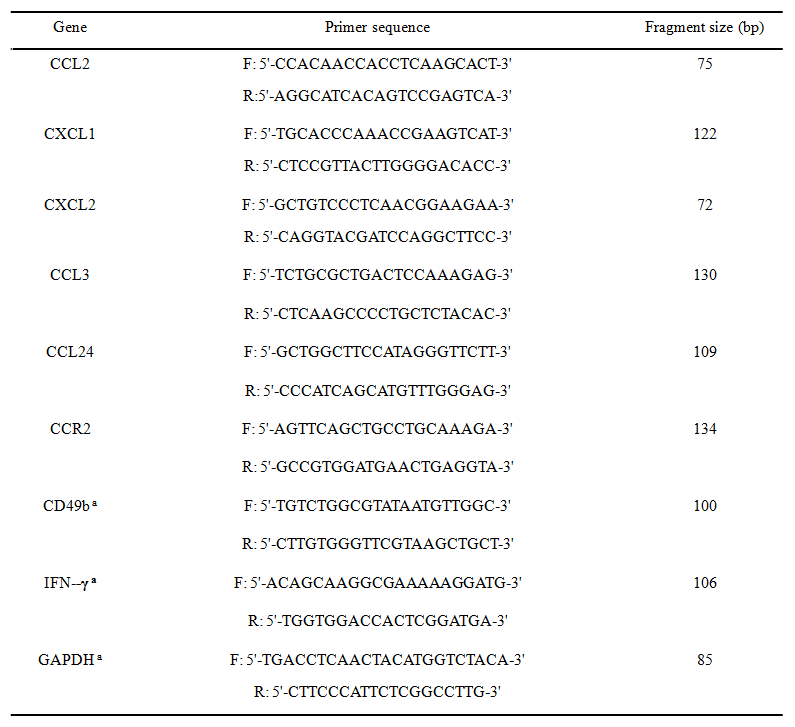


^a^ Primers from reference [11].
